# A one-step multiplex qPCR assay for simultaneous identification and quantification of *Leishmania martiniquensis* and *Leishmania orientalis*/*Leishmania chancei* and detection and quantification of trypanosomatids in clinical samples

**DOI:** 10.1051/parasite/2025030

**Published:** 2025-06-24

**Authors:** Chonlada Mano, Piyapha Hirunpatrawong, Patiya Prasertsilp, Saowalak Kaewmee, Parinda Limprasert, Padet Siriyasatien, Adisak Tantiworawit, Derek Gatherer, Michael D. Urbaniak, Paul A. Bates, Narissara Jariyapan

**Affiliations:** 1 Center of Excellence in Vector Biology and Vector-Borne Disease, Department of Parasitology, Faculty of Medicine, Chulalongkorn University Bangkok 10330 Thailand; 2 Department of Medical and Public Health Secretary, College of Allied Health Sciences, Suan Sunandha Rajabhat University Samut Songkhram 75000 Thailand; 3 Faculty of Medicine, Chulalongkorn University Bangkok 10330 Thailand; 4 Department of Internal Medicine, Faculty of Medicine, Chiang Mai University Chiang Mai 50200 Thailand; 5 Division of Biomedical and Life Sciences, Faculty of Health and Medicine, Lancaster University Lancaster LA1 4YG UK

**Keywords:** qPCR, *Leishmania martiniquensis*, *Leishmania orientalis*, *Leishmania chancei*, *Mundinia*, trypanosomatid

## Abstract

Leishmaniasis is one of the most important zoonotic diseases. Recently, *Leishmania* (*Mundinia*) *martiniquensis*, *Leishmania* (*Mundinia*) *orientalis*, and *Leishmania* (*Mundinia*) *chancei* have been reported as new human pathogens. Trypanosomatids, apart from *Leishmania* spp., such as *Crithidia* spp., are also occasionally capable of infecting humans. Here, a one-step multiplex qPCR assay for the simultaneous identification and quantification of *L. martiniquensis* and *L. orientalis*/*L. chancei* and detection and quantification of trypanosomatids was developed using ITS1 as the molecular target and human *RNase P* as the internal control gene. The assay was evaluated using 44 positive residual DNA samples from leishmaniasis patients and 25 negative DNA samples. Results revealed that the limits of detection (LOD) of the assay for *L. martiniquensis*, *L. orientalis*, and *Crithidia* sp. (CLA-KP1) were 1.699 (0.0255), 1.717 (0.0292), and 1.763 (0.0882) fg/reaction (parasite equivalents/reaction), respectively. The assay had high analytical specificity. The mean Cq values of the intra-assays and inter-assays differed by less than 1, indicating the reliability of the assay. Evaluation results revealed that the assay could identify *L. martiniquensis* and *L. orientalis* in clinical samples with 100% sensitivity and 100% specificity. In conclusion, the ITS1/human *RNase P* multiplex qPCR assay offers a rapid and reliable diagnostic tool for identifying and quantifying *L. martiniquensis* and *L. orientalis*/*L. chancei* and detecting and quantifying trypanosomatids in clinical samples within a single reaction. This assay provides an advancement in the diagnostic capabilities for leishmaniasis and trypanosomatid infections, potentially improving patient management and surveillance efforts.

## Abstract (fr)

La leishmaniose est l’une des zoonoses les plus importantes. Récemment, *Leishmania* (*Mundinia*) *martiniquensis*, *Leishmania* (*Mundinia*) *orientalis* et *Leishmania* (*Mundinia*) *chancei* ont été signalés comme de nouveaux agents pathogènes humains. Outre *Leishmania* spp., les trypanosomatidés, tels que *Crithidia* spp., sont également parfois capables d’infecter l’homme. Un test de qPCR multiplex en une étape pour l’identification et la quantification simultanées de *L. martiniquensis* et de *L. orientalis/L. chancei*, ainsi que pour la détection et la quantification des trypanosomatidés, a été développé en utilisant ITS1 comme cible moléculaire et la RNAase P humaine comme gène de contrôle interne. Le test a été évalué à l’aide de 44 échantillons d’ADN résiduel positifs provenant de patients atteints de leishmaniose et de 25 échantillons d’ADN négatifs. Les résultats ont révélé que les limites de détection (LOD) du test pour *L. martiniquensis*, *L. orientalis* et *Crithidia* sp. (CLA-KP1) étaient respectivement de 1,699 (0,0255), 1,717 (0,0292) et 1,763 (0,0882) fg/réaction (équivalents parasites/réaction). Le test présentait une spécificité analytique élevée. Les valeurs moyennes de Cq des tests intra-tests et inter-tests différaient de moins de 1, ce qui indique la fiabilité du test. Les résultats de l’évaluation ont révélé que le test pouvait identifier *L. martiniquensis* et *L. orientalis* dans des échantillons cliniques avec une sensibilité et une spécificité de 100 %. En conclusion, le test qPCR multiplex ITS1/RNAase P humaine offre un outil de diagnostic rapide et fiable pour l’identification et la quantification de *L. martiniquensis* et *L. orientalis/L. chancei*, ainsi que pour la détection et la quantification des trypanosomatidés dans les échantillons cliniques, en une seule réaction. Ce test constitue une avancée dans le diagnostic de la leishmaniose et des infections par les trypanosomatidés, améliorant potentiellement la prise en charge des patients et la surveillance.

## Introduction

Leishmaniasis is an important vector-borne zoonotic disease that affects an estimated 350 million individuals living in tropical and subtropical areas [72]. It is caused by protozoan parasites of the genus *Leishmania*, which belong to the Trypanosomatidae family and can infect humans as well as several domestic and sylvatic animals [6, 10, 29, 37, 42, 44, 57]. According to the World Health Organization, an estimated 700,000 to 1 million new cases of leishmaniasis occur annually, leading to significant morbidity and mortality, especially in underdeveloped countries [72]. The clinical manifestations of leishmaniasis vary depending on the species and/or strain of *Leishmania* and host immune response. Disease severity ranges from asymptomatic or self-healing cutaneous lesions (cutaneous leishmaniasis, CL) to chronic infections that cause severe tissue destruction in mucocutaneous leishmaniasis (MCL) and can progress to life-threatening visceral leishmaniasis (VL) [72]. Various species of sand flies are well-established vectors of *Leishmania* parasites in the subgenera *Leishmania* and *Viannia*. However, recent reports have documented natural infection of the human pathogen *Leishmania* (*Mundinia*) *martiniquensis* in *Culicoides peregrinus*, suggesting that biting midges may serve as potential vectors of leishmaniasis [22]. Moreover, transmission through blood transfusion may also be possible, as asymptomatic *Leishmania* carriers have been identified among blood donors in several endemic countries, including Brazil and Iran [4, 64]. Recently, at least 21 *Leishmania* species have been identified as human pathogens, primarily belonging to the subgenera *Leishmania* (ten species), *Viannia* (eight species), and the newly identified subgenus *Mundinia* (three species) [62]. The emergence of novel human infective *Leishmania* species of subgenus *Mundinia*, including *L.* (*Mundinia*) *martiniquensis* [8], *L.* (*Mundinia*) *orientalis* [20], and *L.* (*Mundinia*) *chancei* [25], poses an increasing public health and disease control challenge in endemic areas.

Thailand is considered endemic for leishmaniasis, particularly in the northern and southern parts of the country, with confirmed autochthonous cases and a continuously rising number of both symptomatic and asymptomatic cases over the years [5, 27, 53, 63, 66, 72]. This disease is caused by two main protozoan species including *L. martiniquensis* [52] and *L. orientalis* [20]. In an immunocompetent host, *L. martiniquensis* causes VL [52], while *L. orientalis* is mainly responsible for CL [20]. However, VL, diffuse CL, and MCL have been observed in immunocompromised hosts infected with *L. martiniquensis* [66]. Other *Leishmania* species documented in Thailand include *L. donovani* complex, *L. lainsoni*, *L. major*, and *L. infantum* [30, 33]. Recently, amphotericin B-resistant *L. martiniquensis* parasites have also been reported in Thailand [21, 31, 32]. For another *L.* (*Mundinia*) species, *L. chancei* (formerly *L.* “Ghana”), which belongs to a new subgenus and is endemic to West Africa, also causes CL characterized by skin lesions, often ulcers, that can be accompanied by satellite lesions or nodular lymphangitis [26]. The varied clinical manifestations of leishmaniasis caused by *L. martiniquensis*, *L. orientalis*, and *L. chancei* require specific therapeutic and treatment strategies for each species. Furthermore, most of these infection cases are asymptomatic *Leishmania*/HIV co-infections, which pose a major challenge to diagnosing and controlling the disease [33, 67]. The absence of clinical lesions in these individuals, especially in VL/HIV cases, increases the risk of treatment failure/drug resistance and may contribute to the transmission cycle and spread of the disease [2, 39]. International travel and migration by asymptomatic individuals to and from endemic areas can also facilitate the spread of leishmaniasis [65].

Surveys to estimate the prevalence of asymptomatic *Leishmania* infection in endemic areas have been conducted using antibody-based detection methods; however, these methods are unable to distinguish between past and current infections [17, 48]. Currently, no approach exists to define asymptomatic infection, and the only accepted methods to confirm *Leishmania* infection in humans are time-consuming direct parasite examination via light microscopy and parasite cultivation from clinical specimens [17].

Apart from *Leishmania* parasites, other trypanosomatids, such as *Trypanosoma brucei* [51], *T. cruzi* [34], *Crithidia* sp. closely related to *Crithidia fasciculata* [14, 24, 35, 59], and *Leptomonas seymouri* [15, 69], can also infect humans, further complicating diagnosis. *Trypanosoma brucei* and *T. cruzi* are major zoonotic parasites that cause human diseases. *Trypanosoma brucei* causes human African trypanosomiasis (sleeping sickness), primarily in countries in sub-Saharan Africa, while *T. cruzi* causes Chagas disease, primarily in countries in Latin America [34, 51]. Co-infection of *L. major* and *Crithidia* spp. closely related to *C. fasciculata* has been detected in patients from Iran [14, 24]. In Brazil, two clinical isolates, LVH60 and LVH60a, from a 64-year-old man with a fatal visceral leishmaniasis-like illness, are identified as a *Crithidia*-related species, closely related to *C. fasciculata* [35]. Another report is co-infection of *L. infantum* and a *Crithidia*-related species in a case of refractory relapsed visceral leishmaniasis in Brazil [59]. For *Leptomonas*, *L. seymouri* has been reported as co-infection with *L. donovani* in India [15, 69]. Therefore, there is an urgent need to develop a highly sensitive, specific, and quantitative method that can be used not only in Thailand but worldwide to discriminate and quantify the three new *Leishmania* pathogens while, simultaneously detecting and quantifying other human-infected trypanosomatid pathogens in clinical samples.

Quantitative PCR (qPCR)-based approaches have been widely used to detect, identify, and quantify *Leishmania* parasites as the methods are simple, fast, and able to detect low parasite concentrations with broad dynamic range and reduced cross-contamination [13]. Multiplex qPCR assays amplify multiple targets in a single reaction, minimizing the risk of cross-contamination during handling, thereby conserving valuable samples, and reducing the time and cost of running separate qPCR assays. In *Leishmania*, several coding and non-coding regions in the genome have been used as targets in qPCR assays, with the sensitivity and specificity of these assays depending on the copy number of the target region and its uniqueness [1, 16]. These regions include ribosomal DNA (rDNA), spliced-leader (SL) RNA [9, 49], kinetoplast minicircle DNA (mkDNA) or kinetoplast DNA (kDNA), Heat Shock Protein 70 kDa (*HSP70*) [12], arginine permease gene (*AAP3*) [68], glucose-6-phosphate dehydrogenase (G6PD), and DNA polymerase genes [13]. Among the molecular targets, the internal transcribed spacer 1 (ITS1), a non-coding region located on chromosomal DNA between 18S and 5.8S genes, is often selected to design primers and/or probes for detecting *Leishmania* spp. and other trypanosomatids as the region has a high copy number (approximately 20–400 copies) and variable sequences, which can ensure adequate sensitivity and specificity for detection and quantification in qPCR-based assay [13, 18, 28, 70]. Our previous study demonstrated that ITS1-rRNA PCR can detect DNA of *L. martiniquensis* and *L. orientalis* at concentrations as low as 0.01 pg/μL; however, it fails to differentiate between the two species [19]. For the 3′ untranslated region of the heat shock protein 70 gene (*HSP70*-I-3′-UTR) PCR, although it can be used to discriminate between *L. martiniquensis* and *L. orientalis*, its detection limit is less sensitive (1 pg/μL) than that of ITS1-rRNA PCR [19].

So far, a few multiplex qPCR assays have been developed, including an assay to detect *L. infantum* load in sand flies [43], as well as dog, human, and *Leishmania* DNA in sand flies [61], and a novel duplex TaqMan-based quantitative PCR for diagnosing *L. martiniquensis* and *L. orientalis* [54]. However, no multiplex qPCR assays are available for identifying and quantifying the three *Leishmania* (*Mundinia*) pathogens and detecting and quantifying trypanosomatids in a single reaction. Therefore, this study aimed to develop a novel one-step multiplex qPCR assay that could simultaneously identify and quantify the three *Leishmania* (*Mundinia*) species and detect and quantify other trypanosomatids in clinical samples using ITS1 as the molecular target and human *RNase P* as the internal control gene. This developed assay would aid in improving diagnostics, tailoring treatments, and informing public health strategies to effectively control *Leishmania* and trypanosomatid infections, thereby mitigating their impact on human health.

## Materials and methods

### Ethics statement

The study was approved by the ethics committee of the Faculty of Medicine, Chulalongkorn University (COE No. 051/2022) before the study began. The parasites were acquired from the cryopreserved parasite repository and residual DNA samples extracted from anonymized clinical specimens from leishmaniasis patients in northern Thailand where informed consent was waived. Anonymized samples of human DNA were provided from residual specimens sent for diagnosis by the Department of Parasitology, Faculty of Medicine, Chiang Mai University. All DNA samples were sent to the investigator with a number label, which cannot be linked to the identity of the patients and their clinical data. The protocol was conducted in compliance with the CIOMS International Ethical Guidelines for Health-related Research.

### Primers-probe design

Primers and probes were designed using Integrated DNA Technologies, PrimerQuest^TM^ Tool, (https://sg.idtdna.com/pages/tools/primerquest) based on the sequences of the conserved regions of the ITS1 targets of trypanosomatids that are related to human pathogens and available in the GenBank database (https://www.ncbi.nlm.nih.gov/) (Table S1). For the internal control gene, a modified version of the *Homo sapiens* ribonuclease P (human *RNase P*) gene assay (Accession number NM_006413.4) [11] was designed to adjust the melting temperature (Tm) of the multiplex qPCR assay developed in this study (File S1). All primer-probe sets were custom synthesized by LGC Biosearch Technologies (Hoddesdon, UK).

### Parasite strains and gDNAs

Parasites maintained in the Department of Parasitology, Faculty of Medicine, Chulalongkorn University, Thailand, in Schneider’s Insect Medium (SIM) (Sigma-Aldrich, St. Louis, MO, USA), pH 6.8, supplemented with 10% FBS (Life Technologies-Gibco, Grand Island, NY, USA), and 25 μg/mL gentamicin sulfate (Sigma-Aldrich) were used. The parasites included *L*. *martiniquensis* LSCM1 (MHOM/TH/2012/LSCM1), *L*. *martiniquensis* (LSCM1-6), *L*. *martiniquensis* (AmpBRP2i), *L*. *martiniquensis* (LSCM2), *L*. *orientalis* LSCM4 (MHOM/TH/2014/LSCM4), and *Crithidia* sp. (CLA-KP1).

In addition, gDNA of *L*. *orientalis* (PCM2), *Crithidia* sp. (CLA-KP4) *Crithidia* sp. (CLA-TR2) and *Crithidia* sp. (CLA-TR3) stored in 4 °C in the Department of Parasitology, Faculty of Medicine, Chulalongkorn University, and gDNA of *L*. *aethiopica* (LV546), *L*. *amazonensis* (M2269), *L*. *donovani* (LV9), *L*. *infantum* (JPCM5), *L*. *major* (FV1), *L*. *mexicana* (M379), *L*. *tropica* (LV357), *L. braziliensis* (U1096), *L*. *guyanensis* (M4147), *L*. *panamensis* (LS94), *L*. *enrietti*, *L*. *martiniquensis* (LV760), *L*. *chancei*, *L*. *procaviensis*, and *T. brucei* collected in microcentrifuge tubes or on FTA cards from the Department of Biomedical & Life Sciences, Lancaster University, UK were used in this study. The authenticity of the strains and DNA samples was verified by DNA sequencing using 70-IR-D and 70-IR-M primers for all *Leishmania* strains [19, 56], and TRY927F and TRY927R primers for *T. brucei* and *Crithidia* sp. strains [22, 47].

### DNA extraction

DNA of the cultured parasites was extracted using a genomic DNA purification kit (Thermo Fisher Scientific Inc., Waltham, MA, USA), according to the manufacturer’s instructions. For the DNA extraction from FTA cards, a MagPurix Viral/Pathogen Nucleic Acids Extraction Kit A (Zinext, New Taipei City, Taiwan) was used, according to the manufacturer’s instructions. At the final step, DNA was eluted in 100 μL elution buffer and stored at 4 °C and used within 4 weeks. In each batch of DNA extraction, UltraPure^TM^ DNase/RNase-Free Distilled Water (Invitrogen, Thermo Fisher Scientific, Loughborough, UK) was used as a negative control. DNA concentrations of each sample were determined using a NanoDrop spectrophotometer 2000 (Thermo Fisher Scientific Inc., Waltham, MA, USA).

### Conventional PCR (cPCR)

A cPCR method was used to check primer dimerization and specificity of the ITS1 primers designed in Table 1. We performed the cPCR reaction using EconoTaq 2X Master Mix (LGC Biosearch Technologies), in accordance with the manufacturer’s recommendations. Approximately 1 ng of DNA from each sample was used as a template. PCR amplification conditions were as follows: an initial denaturation step at 95 °C for 2 min, followed by 30 cycles at 95 °C for 30 s, 60 °C for 30 s, at 72 °C for 45 s, and final extension at 72 °C for 10 min. Nuclease-free H_2_O was used as a negative control. Expected amplicons of PCR products were separated on 2% agarose gels, stained with ethidium bromide (Thermo Fisher Scientific, Loughborough, UK), and visualized using a GelDoc imaging system (Ultra-Violet Products Ltd., Cambridge, UK).

**Table 1:** Primers and probes for the ITS1 and human *RNase P* targets used in the developed multiplex qPCR assay.

Target	Name of primer-probe set	Sequence	Tm (°C)	Fluorescent Dye/Amplicon size (bp)
ITS1	ITS1-L. mar^a^	Forward: CCACATACACAAACACAGCAATA (Sense)	62	HEX/149
		Probe: GCCAAATGCCGCGCGTATACAG (Sense)	68	
		Reverse: GAGAGAAAGAGCCGTAACGAA (AntiSense)	62	
	ITS1-L. ori/cha^b^	Forward: GGGAGGTGTMTCTCTCTTT (Sense)	60	FAM/146
		Probe: AGATARCGCCTTTCCCACATACACA (Sense)	68	
		Reverse: TACGCYCGGTGTTTATATG (AntiSense)	60	
	ITS1-Tryps^c^	Forward: CGGTGTGTTGTGGATAACGG (Sense)	63	Texas Red/77
		Probe: TAACGTGTCGCGATGGATGACTTGG (Sense)	68	
		Reverse: CTGCGTTCTTCAACGAAATAGGA (AntiSense)	63	
Human *RNase P*	RP-human^d^	Forward: TCAGCATGGCGGTGTTT (Sense)	62	Cy5/81
		Probe: TTCTGACCTGAAGGCTCTGCGC (Sense)	68	
		Reverse: CGGCTGTCTCCACAAGTC (AntiSense)	62	

^a^For detection of *L. martiniquensis*; ^b^For detection of *L. orientalis*/*L. chancei*; ^c^For detection of trypanosomatids; ^d^Internal control

### qPCR optimization and singleplex and multiplex qPCR assays

For qPCR optimization, the concentration of primers and probes and the thermal cycling protocols for the singleplex and multiplex assays were used as recommended in the RapiDxFire qPCR 5X Master Mix GF system user guide (LGC Biosearch Technologies) (File S2). The optimal concentration of the primers and probes and the protocol for the singleplex and multiplex qPCR were as follows. Singleplex qPCR reaction was carried out in a total volume of 10 μL containing 5.75 μL of nuclease-free H_2_O, 2 μL of RapiDxFire qPCR 5X Master Mix GF, 0.25 μL of 40X each primers-probe (125 nM/50 nM), and 2.0 μL of template DNA. Multiplex qPCR was carried out in a total volume of 10 μL, following the method of the singleplex assay, with the exception that the volume of nuclease-free H_2_O was adjusted to 5 μL and 1 μL of a 40X primers-probe mix (125 nM/50 nM for each primer-probe) was utilized. The qPCR amplification was performed in a CFX96 (Bio-Rad Laboratories, Foster City, CA, USA) using the following thermal cycling protocol: 1 cycle of polymerase activation at 95 °C for 5 min, followed by 45 cycles of PCR at 95 °C for 15 s, and 60 °C for 1 min. Positive controls consisted of DNA extracted from cultured promastigotes of *L*. *martiniquensis* and *L*. *orientalis*, whereas a master mix without DNA was used as no template control (NTC). TaqMan^TM^ Control Genomic DNA (human) (Applied Biosystems^TM^, Thermo Fisher Scientific Baltics UAB, Vilnius, Lithuania) targeting Human *RNase P* gene was used as both an internal control and a negative control in the multiplex qPCR assay. Reactions were performed in triplicate, and any inconsistent or undetermined results among the replicates were regarded as negative.

### Standard curve of singleplex and multiplex qPCR assays

DNA of *L. martiniquensis* (10^7^ fg/μL ≈ 1.5 × 10^5^ parasites), *L. orientalis* (10^7^ fg/μL ≈ 1.7 × 10^5^ parasites), and *Crithidia* sp. (CLA-KP1) (10^7^ fg/μL ≈ 5 × 10^5^ parasites) extracted from culture were used to prepare serial dilutions for singleplex and multiplex qPCR assays. The DNA standards were serially diluted into 10-fold dilutions ranging from 10^6^ to 1 fg/μL (seven serial dilutions) by mixing with the TaqMan^TM^ Control Genomic DNA (human) in a proportion of 1:9. We diluted the parasite DNA with human DNA to confirm that no background problem would be caused by human DNA in clinical specimens, and we also used human DNA as the internal control of the assay.

To obtain a standard curve, in each reaction of each assay, 2.0 μL of the serially diluted DNA of each parasite species were used, thus, the DNA quantities used in each assay were 2 × 10^6^–2 fg/reaction. Each assay was performed in three independent experiments in triplicate. Standard curves were established by plotting cycle threshold (Cq) against log_10_ parasite DNA quantity (fg/reaction) and linear regression was done, enabling the determination of the correlation coefficient (*R*^2^). The amplification efficiencies (*E*) were calculated using the equation *E* = (10^(−1/slope) − 1^) × 100% [45]. *R*^2^ values of ≥0.99 were considered desirable. A reaction efficiency between 90% and 110% was considered acceptable for the qPCR.

### Analytical sensitivity and specificity of the multiplex qPCR assay

After multiplex qPCR protocol standardization, the cut-off value was defined by means of receiver operating characteristic (ROC) analysis to obtain the lowest Cq with 100% specificity and the highest sensitivity [7]. The limit of detection of the multiplex qPCR was assessed using curve-fitting methods proposed and defined by Klymus *et al*. [23]. Serial dilutions with the TaqMan^TM^ Control Genomic DNA (human) of the *L. martiniquensis*, *L. orientalis*, and *Crithidia* sp. (CLA-KP1) DNA in quantities of 5, 2, 1.5, 1, 0.5, and 0.2 fg/reaction were used. The qPCR of each dilution was performed in four independent experiments in triplicate (12 tests). The limit of detection with 95% confidence in the detected probability (LOD95) was determined by statistical probit analysis (non-linear regression model) using GraphPad Prism version 10.2.3 software (GraphPad Software; San Diego, CA, USA).

To evaluate the specificity of the designed primer probes, artificial DNA mixtures (1:1) of human DNA mixed with DNA of known species or strains of *Leishmania*, *Trypanosoma*, and *Crithidia* sp. (above) were used as a template. The multiplex qPCR results of the known non-target species were used to assess analytical specificity. We used the DNA of *Plasmodium vivax*, *P. falciparum*, and *Mycobacterium tuberculosis* as a template for known non-target species, which are pathogens commonly found in blood samples. All tests were performed in triplicate.

### Intra-assay repeatability and inter-assay reproducibility of the multiplex qPCR assay

To evaluate the repeatability and reproducibility of the multiplex qPCR assay, three parasite DNA quantities (2 × 10^5^, 2 × 10^3^, and 2 fg/reaction) of each DNA standard of *L*. *martiniquensis*, *L*. *orientalis*, and *Crithidia* sp. (CLA-KP1) were used as templates. In each dilution, the intra-assay test was performed in triplicate within the same run. For the inter-assay test, four separate experiments were conducted independently on different days (Days 1–4) within a week. Then, the coefficients of variation (%CV = SD/mean × 100) for Cq values were compared to assess the repeatability and reproducibility of the assay. Statistical measures, including mean, standard deviation, and coefficient of variation (CV), were calculated using Microsoft Excel (Microsoft Corp., Redmond, WA, USA). The low variability in the mean Cq values and a %CV of less than 5% indicated that the assays demonstrated reliable performance.

### Evaluation of the multiplex qPCR assay

A total of 69 residual DNA samples from northern Thailand stored at 4 °C were used to evaluate diagnostic sensitivity and specificity. We used the *HSP70-I*-3′-UTR PCR as a reference assay for identification of *L. martiniquensis* and *L. orientalis* in the samples [19]. The samples included 44 DNA samples (42 of *L. martiniquensis* and 2 of *L. orientalis*) extracted from clinical specimens of leishmaniasis patients and 25 DNA samples extracted from human blood samples which were negative by PCR for *Leishmania*.

By comparing the results of the multiplex qPCR assay with the *HSP70*-*I*-3′-UTR PCR method, we evaluated the sensitivity and specificity in a two-by-two table. The number of true positive (TP) samples correctly diagnosed by both assays was divided by the total number of true positive (TP) and false negative (FN) samples to calculate the diagnostic sensitivity of each species. The diagnostic specificity of each species was calculated by dividing the number of true negative (TN) samples that were correctly diagnosed by both assays by the total number of true negative (TN) and false positive (FP) samples. The number of true positive (TP) samples was divided by the total number of true positive (TP) and false positive (FP) samples to calculate the positive predictive value (PPV), or precision. The number of true negative (TN) samples was divided by the total number of true negative (TN) and false negative (FN) samples to calculate the negative predictive value (NPV) [41]. Cohen’s kappa statistic value [36] was used to determine the level of agreement between the multiplex qPCR and the reference assay for each species.

## Results

### Designed primers and probes

**Figure 1:**
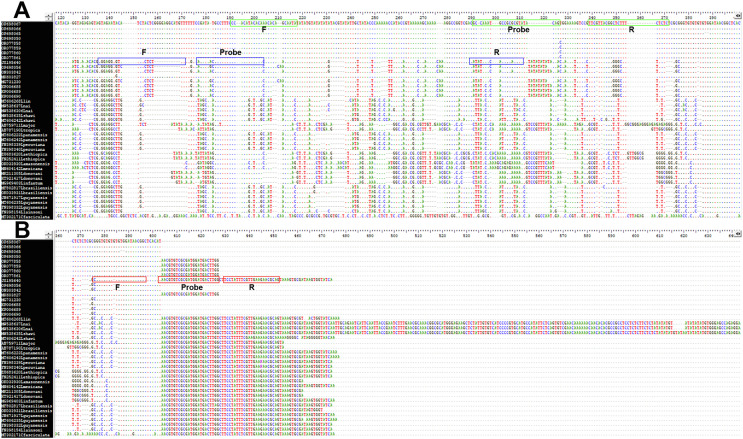
Alignment of the conserved regions using Crustal analysis of (A) ITS1 target for *Leishmania martiniquensis* (green boxes) and ITS1 target for *L. orientalis*/*L. chancei* (blue boxes) and (B) ITS1 target for trypanosomatids (red boxes) used to design primers (F, R) and probes (Prob).

ITS1 sequences were used as targets for designation of the primers and probes to identify *L. martiniquensis* and *L. orientalis*/*L. chancei* and detect trypanosomatids (Table 1, Fig. 1). In the cPCR, the primers, ITS1-L. mar and ITS1-L. ori/cha successfully amplified only the gDNA of *L. martiniquensis* and *L.* orientalis/*L. chancei*, respectively. The ITS1-Tryps primers amplified the gDNA of trypanosomatids (*Leishmania* spp., *T*. *brucei*, and *Crithidia* sp.) without primer dimerization (File S3). No PCR amplicons of *P. vivax*, *P. falciparum*, or *M. tuberculosis* were observed.

### Standard curve of the singleplex and multiplex qPCR assays

**Figure 2:**
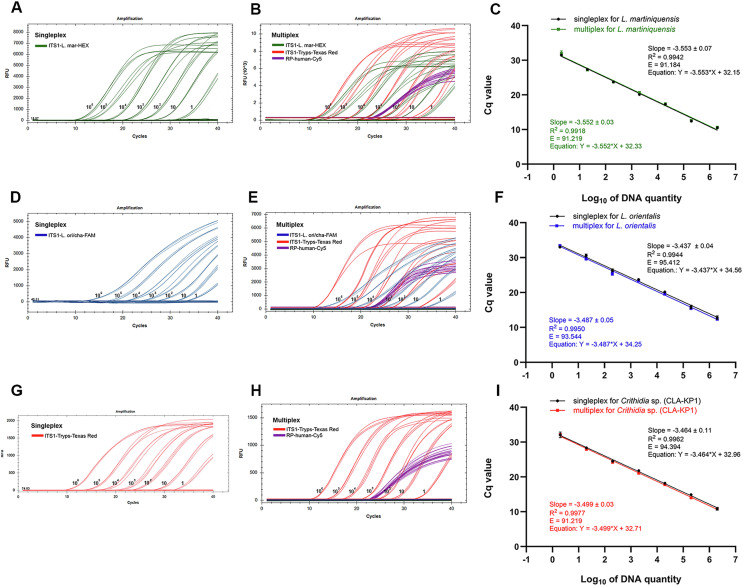
Representatives of amplification plots of the singleplex and multiplex qPCR assays and standard curves of the singleplex and multiplex qPCR of *L. martiniquensis* (A, B, C), *L. orientalis* (D, E, F, or *Crithidia* sp. (CLA-KP1) (G, H, I).

To investigate the detection range of the assays, the DNA standards of *L*. *martiniquensis*, *L*. *orientalis*, and *Crithidia* sp. (CLA-KP1) were serially diluted 10-fold and used as templates in the singleplex and multiplex qPCR. The Cq values were plotted against log_10_ DNA quantity, and standard curves of each target were obtained. No significant differences in sensitivity and specificity in amplifying expected products were observed between the singleplex and multiplex qPCR standard curves. The *R*^2^, *E*, and slope were determined. The *R*^2^ of the standard curves was greater than 0.99 and the *E* values of each target were between 91% and 95%, which were considered acceptable (Fig. 2).

### Analytical sensitivity and specificity of the multiplex qPCR assay

Based on the ROC curve analysis, the cut-off points for *L. martiniquensis*, *L. orientalis*, and *Crithidia* sp. (CLA-KP1) DNA detection were Cq of 38.04, 38.92, and 38.11, respectively (File S4). The LOD was determined through the serial dilutions of known concentrations of *L. martiniquensis*, *L. orientalis*, and *Crithidia* sp. (CLA-KP1) DNA, and the positive detection rates of each dilution were determined. The LOD95 was calculated to be 1.699 fg/reaction (0.0255 parasite equivalents/reaction), 1.717 fg/reaction (0.0292 parasite equivalents/reaction), and 1.763 fg/reaction (0.0882 parasite equivalents/reaction) for *L. martiniquensis*, *L. orientalis*, and *Crithidia* sp. (CLA-KP1), respectively (File S5).

The analytical specificity was assessed by the interference of *P. vivax*, *P. falciparum*, and *M. tuberculosis* on the detection results of the assay. No amplification was found for the DNA of these samples, except for the human DNA in the *P. vivax* and *P. falciparum* DNA samples as they were extracted from clinical samples. This result indicates the high analytical specificity of the multiplex qPCR assay (Table 2).

**Table 2:** Analytical specificity of the multiplex qPCR assay.

DNA samples	Quantification cycle (Cq) (Mean ± SD)			
	ITS1-L. mar-HEX	ITS1-L. ori/cha-FAM	ITS1-Tryps- Texas Red	RP-human-Cy5
*L. martiniquensis* (LV760) + human DNA^a^	12.77 ± 0.12	ND^d^	11.36 ± 0.14	25.58 ± 0.12
*L. martiniquensis* (LSCM1) + human DNA^a^	12.77 ± 0.05	ND	12.47 ± 0.08	25.45 ± 0.09
*L. martiniquensis* (LSCM1-6) + human DNA^a^	11.11 ± 0.11	ND	14.20 ± 0.04	25.85 ± 0.06
*L. martiniquensis* (AmpBRP2i) + human DNA^a^	11.12 ± 0.15	ND	13.12 ± 0.08	25.63 ± 0.19
*L. martiniquensis* (LSCM2) + human DNA^a^	13.49 ± 0.02	ND	13.53 ± 0.11	25.89 ± 0.15
*L. orientalis* (LSCM4) + human DNA^a^	ND	11.31 ± 0.14	13.78 ± 0.01	25.52 ± 0.02
*L. orientalis* (PCM2) + human DNA^a^	ND	16.32 ± 0.21	11.12 ± 0.04	25.18 ± 0.12
*L. chancei* + human DNA^a^	ND	16.44 ± 0.06	17.63 ± 0.04	25.65 ± 0.20
*L. aethiopica* + human DNA^a^	ND	ND	10.64 ± 0.12	25.39 ± 0.10
*L. amazonesis* + human DNA^a^	ND	ND	13.24 ± 0.21	25.30 ± 0.06
*L. donovani* + human DNA^a^	ND	ND	16.78 ± 0.03	25.74 ± 0.08
*L. infantum* + human DNA^a^	ND	ND	15.28 ± 0.01	25.24 ± 0.10
*L. major* + human DNA^a^	ND	ND	14.46 ± 0.21	25.40 ± 0.04
*L. mexicana* + human DNA^a^	ND	ND	12.71 ± 0.16	25.19 ± 0.08
*L. tropica* + human DNA^a^	ND	ND	13.51 ± 0.16	25.18 ± 0.14
*L. braziliensis* + human DNA^a^	ND	ND	15.06 ± 0.02	25.87 ± 0.02
*L. guyanesis* + human DNA^a^	ND	ND	14.13 ± 0.05	25.34 ± 0.18
*L. panamensis* + human DNA^a^	ND	ND	15.78 ± 0.14	25.22 ± 0.11
*T. brucei* + human DNA^a^	ND	ND	25.31 ± 0.05	24.49 ± 0.05
*Crithidia* sp. (CLA-KP1) + human DNA^a^	ND	ND	13.27 ± 0.19	25.13 ± 0.21
*Crithidia* sp. (CLA-KP4) + human DNA^a^	ND	ND	15.27 ± 0.15	25.42 ± 0.11
*Crithidia* sp. (CLA-TR2) + human DNA^a^	ND	ND	14.11 ± 0.12	25.39 ± 0.14
*Crithidia* sp. (CLA-TR3) + human DNA^a^	ND	ND	14.74 ± 0.22	25.40 ± 0.01
*P*. *vivax*^b^	ND	ND	ND	25.30 ± 0.16
*P. falciparum* ^b^	ND	ND	ND	25.45 ± 0.11
*M*. *tuberculosis*^c^	ND	ND	ND	ND

^a^Artificial mixture; ^b^Clinical sample; ^c^Culture sample; ^d^ND = not detected.

### Repeatability and reproducibility of the multiplex qPCR assay

A total of 12 tests of each parasite species were conducted and the mean Cq and %CV values are shown in Table 3. The %CV values of intra- and inter- assays were in the range of 0.23–1.34% and 0.19–2.50%, respectively. The difference between mean Cq values of the intra- and inter-assays was less than 1, suggesting that the multiplex qPCR assay is reliable.

**Table 3:** Repeatability and reproducibility analysis of the multiplex RT-qPCR assay.

DNA sample	DNA quantity (fg/reaction)	Intra assay					Inter assay					
		Cq value^a^			Mean ± SD^b^	%CV^c^	Cq value				Mean ± sd	%CV
		Replicate 1	Replicate 2	Replicate 3			Day 1	Day 2	Day 3	Day 4		
*L. martiniquensis*	2 × 10^5^	12.88	12.78	12.89	12.85 ± 0.06	0.47	12.85	12.92	12.21	12.68	12.67 ± 0.32	2.50
	2 × 10^3^	20.60	20.69	20.62	20.64 ± 0.05	0.23	20.64	20.92	20.50	20.64	20.67 ± 0.18	0.85
	2 × 10^0^	32.28	32.56	32.64	32.49 ± 0.19	0.58	32.49	32.24	31.13	31.86	31.93 ± 0.59	1.86
*L. orientalis*	2 × 10^5^	15.15	15.35	15.55	15.35 ± 0.20	1.30	15.35	15.70	15.25	15.60	15.47 ± 0.21	1.35
	2 × 10^3^	23.34	22.94	23.22	23.17 ± 0.21	0.89	23.17	23.35	23.42	23.23	23.29 ± 0.11	0.49
	2 × 10^0^	33.21	33.77	33.62	33.53 ± 0.29	0.86	33.53	33.66	33.13	33.39	33.43 ± 0.23	0.69
*Crithidia* sp. (CLA-KP1)	2 × 10^5^	13.84	13.68	13.92	13.81 ± 0.12	0.88	13.81	14.05	14.20	14.02	14.02 ± 0.16	1.14
	2 × 10^3^	21.37	20.81	21.15	21.11 ± 0.28	1.34	21.11	21.12	20.98	21.23	21.11 ± 0.10	0.48
	2 × 10^0^	32.18	32.47	31.73	32.13 ± 0.37	1.16	32.13	32.27	32.19	32.17	32.19 ± 0.06	0.19

^a^Cq = Cycle threshold value; ^b^Mean ± SD = Mean ± standard deviation; ^c^%CV = Coefficient of variation = (SD/mean) × 100.

### Evaluation of the multiplex qPCR assay in residual DNA samples

The diagnostic sensitivity and specificity of the multiplex qPCR assay were evaluated using 69 residual DNA samples: 44 positive samples (42 *L. martiniquensis* and 2 *L. orientalis*) and 25 negative samples (Table S2). The 42 *L. martiniquensis* samples and 2 *L. orientalis* samples were identified by both the multiplex qPCR and the *HSP70*-I-3′-UTR PCR methods. No false negatives were found, indicating that the multiplex qPCR has a diagnostic sensitivity of 100% for these two species.

For diagnostic specificity, both multiplex qPCR and *HSP70*-I-3′-UTR PCR methods gave negative results with all 25 negative samples, indicating that this assay has a diagnostic specificity of 100% for *L. martiniquensis* and *L. orientalis*. As *L. martiniquensis* and *L. orientalis* are trypanosomatids, all 44 positive samples were also true positive for trypanosomatids. The diagnostic sensitivity and specificity of the qPCR assay for detection of trypanosomatids were both 100%. Also, the multiplex qPCR assay demonstrated PPV and NPV of 100% and a perfect agreement (kappa = 1.0) with the reference assay for identification of both *Leishmania* species and detection of trypanosomatids (Table 4). The multiplex qPCR assay detected parasites ranging from 0.20–1822.01 and 23.33–50.84 parasite equivalents/reaction in the clinical samples of *L. martiniquensis* and *L. orientalis*, respectively (Table S2).

**Table 4:** Evaluation results of the multiplex qPCR assay compared to the *HSP70*-I-3′-UTR PCR assay for identification of *L. martiniquensis* and *L. orientalis* and detection of trypanosomatid.

Target	Multiplex qPCR	*HSP70-I*-3′-UTR PCR		Total	
		Positive (TP^a^)	Negative (TN^b^)		
*L. martiniquensis*	Positive (TP)	42	0	42	^e^PPV = TP/(TP + FP) = 100%
	Negative (TN)	0	27	27	^f^NPV = TN/(TN + FN) = 100%
	Total	42	27	69	Kappa = 1.0
		Sensitivity = TP/(TP + FN^c^) = 100%	Specificity = TN/(TN + FP^d^) = 100%		
*L. orientalis*	Positive (TP)	2	0	2	PPV = 100%
	Negative (TN)	0	67	67	NPV = 100%
	Total	2	67	69	Kappa = 1.0
		Sensitivity = 100%	Specificity = 100%		
Trypanosomatids	Positive (TP)	44	0	44	PPV = 100%
	Negative (TN)	0	25	25	NPV = 100%
	Total	44	25	69	Kappa = 1.0
		Sensitivity = 100%	Specificity = 100%		

^a^TP = true positive; ^b^TN = true negative; ^c^FN = false negative = 0; ^d^FP = false positive = 0; ^e^PPV = positive predictive value; ^f^NPV = negative predictive value.

## Discussion

Most previous studies have focused on developing singleplex qPCR assays for pan-genus *Leishmania* detection using highly sensitive targets, such as mkDNA or kDNA, spliced-leader (SL) RNA [12, 49], the arginine permease gene (*AAP3*) [68], *HSP70* gene, and 18S rDNA, which characterize the *Leishmania* genus [12, 55, 71, 73]. In recent years, several qPCR assays have been developed to detect *Leishmania* spp. DNA in clinical samples. For instance, Eberhardt *et al*. [9] developed an SL-RNA qPCR assay that demonstrated exceptional analytical sensitivity, detecting 0.005 and 0.002 parasites per mg of liver and spleen tissue, respectively. This SL-RNA qPCR assay is equally effective in detecting *L. infantum*, *L. donovani*, *L. tropica*, *L. major*, *L. mexicana*, *L. panamensis*, *L. guyanensis*, and *L. braziliensis*. In another study, qPCR assays targeting the *HSP70* and 18S rDNA genes of *Leishmania* spp. in multiplex with the human *RNAse P* gene have been developed and validated. The assays can detect up to 0.01 parasite equivalents/reaction and up to 0.1 parasite equivalents/reaction for the *HSP70* target. The assays could detect DNA from *L. amazonensis*, *L. guyanensis*, *L. panamensis*, and *L. braziliensis* [12]. Although these assays have shown excellent sensitivity and specificity for detecting most studied *Leishmania* spp., no species discrimination is possible.

Here, we successfully developed a novel one-step multiplex qPCR assay that simultaneously identified and quantified *L. martiniquensis* and *L. orientalis*/*L. chancei* parasites and detected and quantified other trypanosomatids in clinical samples, using ITS1 as the molecular target and human *RNase P* as the internal control gene. The developed assay provided high diagnostic values of 100% sensitivity and high analytical specificity, at a concentration of approximately 1.7 (0.03) fg/reaction (parasite equivalents/reaction) for the LOD95. In addition, the repeatability and reproducibility analysis of the multiplex qPCR assay revealed that the test exhibited good reproducibility across different testing days, with no inconclusive results or statistical differences between replicates. These findings indicate that the assay is reliable for clinical diagnosis and appropriate for clinical application in detection and identification of the new species without the need for parasite isolation and cultivation. In the case of weak positives or near-threshold results, sequencing of the amplified DNA is required to confirm the presence of the target sequence (https://www.epa.gov/sites/default/files/2015-07/documents/epa-qaqc-pcr.pdf).

Recently, the novel duplex TaqMan-based qPCR for the diagnosis of *L. martiniquensis* and *L. orientalis* using the ITS1 and the heat shock protein 70 (type I) intergenic region (*HSP70-I* IR) as targets has demonstrated that the LOD of *L. martiniquensis* and *L. orientalis* is approximately 1 copy per reaction [55]. However, this could not be compared to our study, as their study used a standard plasmid as a template for assay analysis. In our study, human DNA was used to dilute the parasite DNA to confirm that no background problem was caused by human DNA in clinical specimens, thereby indicating true sensitivity of our multiplex qPCR assay for diagnosis.

The inability of the developed multiplexed qPCR assay to distinguish between *L. orientalis* and *L. chancei* should not limit its clinical utility due to the geographical separation of these species, as *L. orientalis* is endemic only in Thailand and *L. chancei* is endemic only in West Africa [20, 25, 26, 33, 60]. However, only two *L. orientalis* and one *L. chancei* true-positive samples were available in this study. Therefore, a larger and geographically diverse cohort, including *L. orientalis* and *L. chancei* samples from different endemic regions, would be necessary for robust validation in the future.

Like pan-genus *Leishmania* detection qPCR assays [9, 49], our developed multiplex qPCR assay has a limitation, *i.e.*, that mixed infection of other *Leishmania* spp. or trypanosomatids cannot be excluded. Since the ITS1-Tryps-Texas Red primer-probe set was designed to detect all trypanosomatids, it could also amplify the DNA of *L. martiniquensis*, *L. orientalis*, and *L. chancei*. Thus, physicians in the synanthropic area where multiple species are endemic should be aware of this limitation. However, the advantage of this multiplex qPCR assay is its ability to screen for other *Leishmania* spp. and trypanosomatid infections in the same run. Overall, the developed multiplex qPCR assay serves its intended purpose, *i.e.*, identification and quantification of emerging leishmaniasis by *L. martiniquensis* and *L. orientalis*/*L. chancei* parasites and detection and quantification of trypanosomatid infection in humans in a single reaction.

While *L. orientalis* has only been reported in Thailand [3], the epidemiology of *L. martiniquensis* reveals a global distribution, with human cases reported in Martinique [8], Thailand (reviewed by [27]), and Myanmar [46], as well as cases in horses in the United States [38, 57], Germany [44], Switzerland [44], and Brazil [37], and in cows in Switzerland [29]. In addition, asymptomatic leishmaniasis cases caused by *L. orientalis* and *L. martiniquensis* have been detected in both the northern and southern regions of Thailand [33, 67]. In a southern province in Thailand, an asymptomatic *Leishmania* infection has been detected among blood donors with a prevalence of 19%, with *L. martiniquensis* being the predominant species [50]. Investigations regarding whether the asymptomatic individuals could potentially harbor and transmit the parasite through blood products should be conducted, using highly sensitive and reliable methods such as qPCR due to very low blood parasitemia. Furthermore, inspection of the *Leishmania* infection results, from blood donors, should be performed attentively as gold-standard methods, *i.e.*, Giemsa staining and cultivation cannot be used to identify parasites in asymptomatic carriers [40]. Given the concern over *Leishmania* transmission via blood transfusion, our developed multiplex qPCR assay provides an additional promising tool for screening blood products for *Leishmania* DNA. Furthermore, the multiplex qPCR assay would be useful in public health surveillance for the detection of asymptomatic carriers.

Besides the *Leishmania* parasites, the multiplex qPCR assay can detect trypanosomatids that infect humans, such as *T. brucei*, facilitating surveillance, monitoring, and management of the diseases. This assay would significantly impact disease management by providing a rapid, accurate, and species-specific diagnosis, thereby enabling prompt treatment and appropriate therapy. The potential spread of *Leishmania* parasites to non-endemic regions could be due to increased population migration, international travel activities of humans and animal hosts, growth and spread of vector populations, and increased asymptomatic cases [58]. Thus, our developed multiplex qPCR assay could be applied to all endemic areas with leishmaniasis and other trypanosomatid infections worldwide, not limited to Thailand. However, in the future, additional field validation should be performed, exploring the utility of the assay with various clinical samples in different endemic settings, to confirm the actual value of this tool.

## Conclusion

The ITS1/human *RNase P* multiplex qPCR assay for the simultaneous identification and quantification of *L. martiniquensis*, *L. orientalis*/*L. chancei* parasites, and detection and quantification of other trypanosomatids in clinical samples was developed, with high diagnostic values of 100% sensitivity and high specificity. The assay detected a minimum of 0.0255 parasite equivalents/reaction for *L. martiniquensis*, 0.0292 parasite equivalents/reaction for *L. orientalis*, and 0.0882 parasite equivalents/reaction for *Crithidia* sp. (CLA-KP1). This newly developed multiplex qPCR assay offers a rapid, precise, and reliable diagnostic tool for future applications in diagnosing the three new *Leishmania* species and detecting and quantifying other trypanosomatid parasites. Rapid and effective diagnosis of leishmaniasis would benefit patients to receive appropriate therapy and prompt treatment to reduce possible complications. The developed multiplex qPCR assay would also benefit large-scale screening and surveillance programs in detecting *L. martiniquensis*, *L. orientalis*, and trypanosomatid parasites in asymptomatic individuals, especially people living with HIV and blood donors in Thailand and worldwide.

## Figures and Tables

**File S1.**
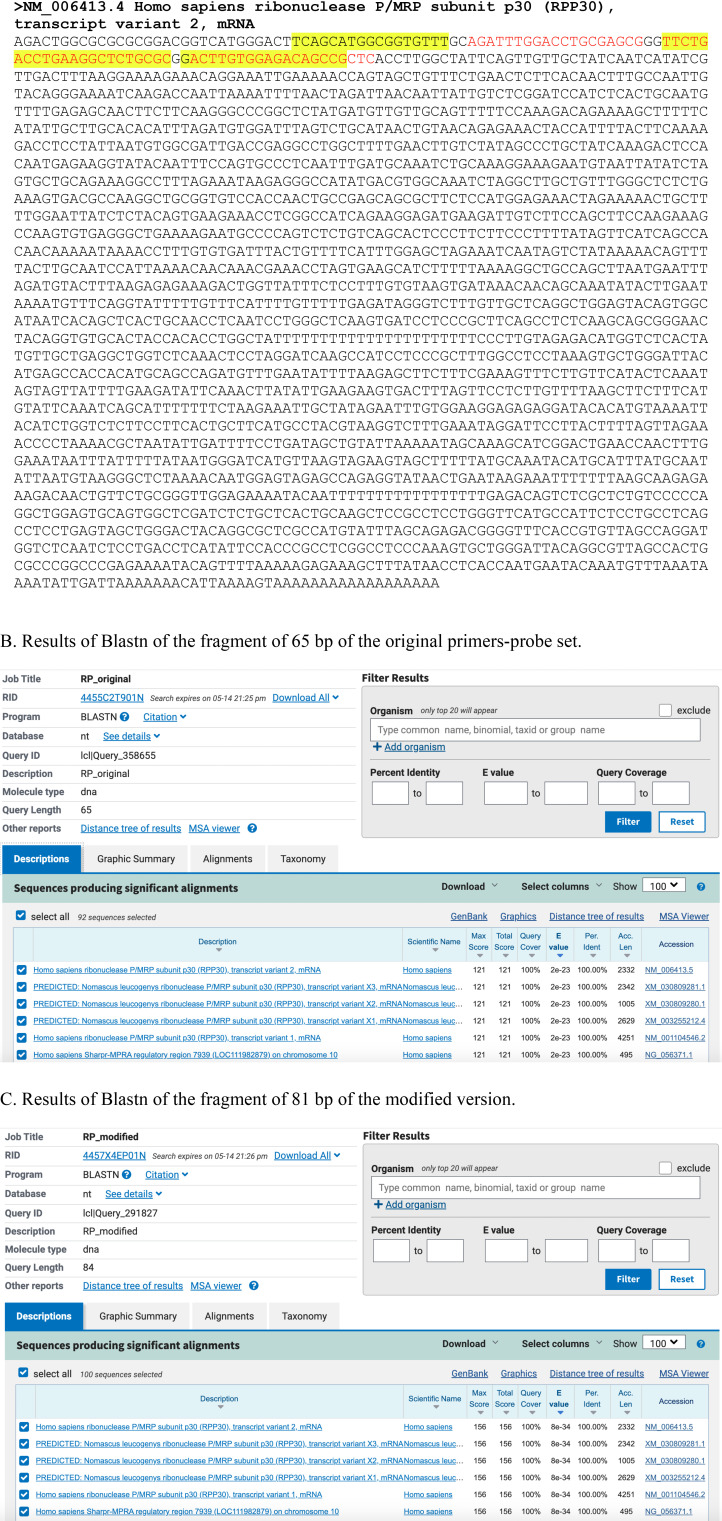
A. The original version of the primers-probe set of *Homo sapiens* ribonuclease P (RP forward: AGATTTGGACCTGCGAGCG; RP probe: TTCTGACCTGAAGGCTCTGCGCG; RP reverse: GAGCGGCTGTCTCCACAAGT) designed by Fan *et al*. [11] is shown in red letters and the modified version (RP-human forward: TCAGCATGGCGGTGTTT; RP-human probe: TTCTGACCTGAAGGCTCTGCGC; RP-human reverse: CGGCTGTCTCCACAAGTC) is in yellow highlight. Both sets were designed from the reference sequence (GenBank accession number): NM_006413.4. B. Results of Blastn of the fragment of 65 bp of the original primers-probe set. C. Results of Blastn of the fragment of 81 bp of the modified version.

**File S2.**
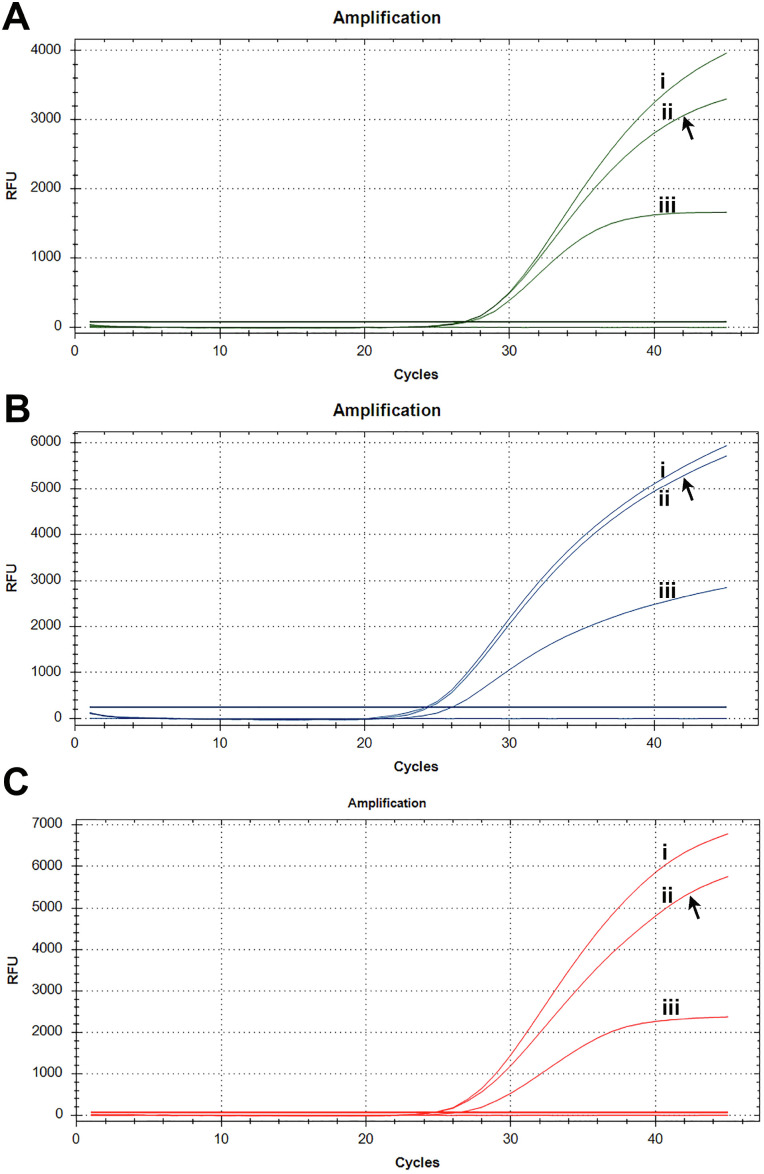
Representative of optimization for the multiplex qPCR assay using primers-probe concentration for each target at (i) 250 nM primers/100 nM probe, (ii) 125 nM primers/50 nM probe, and (iii) 62.5 nM primers/25 nM probe and 102 fg/reaction for (A) *L. martiniquensis*, (B) *L. orientalis*, and (C) *Crithidia* sp. (CLA-KP1) as templates. The qPCR reactions were conducted according to the recommended thermal cycling protocol: 1 cycle of polymerase activation at 95 °C for 5 min, followed by 45 cycles of PCR at 95 °C for 15 s, and 60 °C for 1 min.

**File S3.**
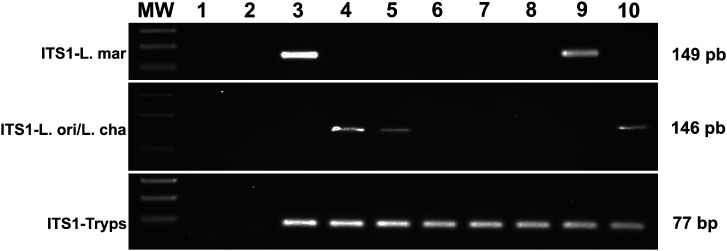
PCR products of the ITS1 fragment of 149 bp using ITS1-L. mar primers, the ITS1 fragment of 146 bp using ITS1-L. ori/cha primers, and the ITS1 conserved fragment of 77 bp using ITS1-Tryps primers. Lanes: MW = Molecular weight markers; 1 = Negative control; 2 = human DNA; 3 = *L. martiniquensis*; 4 = *L. orientalis*; 5 = *L. chancei*; 6 = *L. infantum*; 7 = *L. braziliensis*; 8 = *Crithidia* sp. (CLA-KP1); 9 = *L. martiniquensis* + human DNA; 10 = *L. orientalis* + human DNA.

**File S4.**
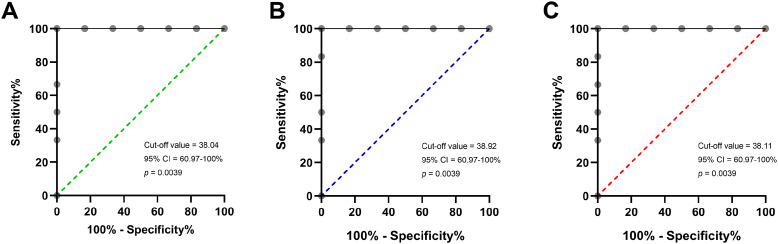
Receiver operating characteristic (ROC) analysis for cut-off estimation for (A) *L. martiniquensis*, (B) *L. orientalis*, and (C) *Crithidia* sp. (CLA-KP1).

**File S5.**
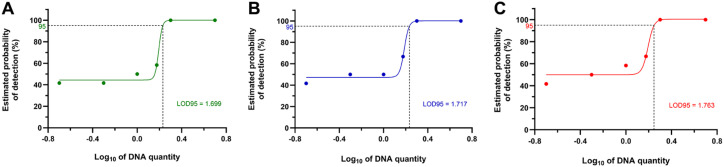
Limit of detection (LOD95) of the multiplex qPCR for (A) *L. martiniquensis*, (B) *L. orientalis*, and (C) *Crithidia* sp. (CLA-KP1) was estimated by fitting a logistic regression model with detection results. Each dot indicates an actual detection rate at each dilution. The dashed line in the figure indicates the dilution ratio corresponding to a 95% probability.

## Supplementary material

The supplementary materials of this article are available at https://www.parasite-journal.org/10.1051/parasite/2025030/olm.

*Table S1.* Selected sequences of ITS1 targets of trypanosomatids that were used to design the primers and probes in this study. *Table S2.* Clinical diagnosis in the 69 residual DNA samples: 44 positive (42 for *L. martiniquensis* and 2 for *L. orientalis*) samples extracted from clinical specimens of leishmaniasis patients and 25 negative samples using the multiplex qPCR assay.

## Notes

**Cite this article as**: Mano C, Hirunpatrawong P, Prasertsilp P, Kaewmee S, Limprasert P, Siriyasatien P, Tantiworawit A, Gatherer D, Urbaniak MD, Bates PA & Jariyapan N. 2025. A one-step multiplex qPCR assay for simultaneous identification and quantification of *Leishmania martiniquensis* and *Leishmania orientalis/Leishmania chancei* and detection and quantification of trypanosomatids in clinical samples. Parasite **32**, 37. https://doi.org/10.1051/parasite/2025030.
