# Supplementary material for: A one-step multiplex qPCR assay for simultaneous identification and quantification of Leishmania martiniquensis and Leishmania orientalis/Leishmania chancei and detection and quantification of trypanosomatids in clinical samples
Source: Parasite. 2025 Jun 24;32:37. doi: 10.1051/parasite/2025030 (PMC12187069; doi:10.1051/parasite/2025030)
Supplement: Supplementary file 1 — Table S1. Selected sequences of ITS1 targets of trypanosomatids that were used to design the primers and probes in this study. [file parasite-32-37-s1.pdf]

Table S1. Selected sequences of ITS1 targets of trypanosomatids that were used to design the primers and probes in this study.

Trypanosomatids	Genbank accession number
L. martiniquensis	OP698065.1, OP698066.1, OP698067.1, OP698050.1, OR077858.1, OR077859.1, OR077860.1, OR077861.1, MK603827.1
L. orientalis	OP698056.1, ON303842.1, MG731230.1, JX195640.1
L. chancei	KP006688.1, KP006689.1, KP006690.1
L. amazonensis	OK033930.1
L. aethiopica	EU683620.1, FN252411.1
L. mexicana	MN604142.1
L. lindenbergi	MT606268.1
L. donovani	MK211305.1, KT921417.1
L. naiffi	MW538636.1, MW538637.1
L. infantum	MG969403.1
L. shawi	MW538635.1, MT606242.1
L. braziliensis	MF802817.1, OK033931.1
L. major	AB759711.1
L. guyanensis	JN671917.1, MT606235.1, FN398332.1
L. tropica	AB787190.1
L. panamensis	MT606223.1, MT606243.1
L. peruviana	FN398339.1, FN398340.1
L. lainsoni	FN398154.1
Cr. fasciculata	MT302171.1
