# Supplementary material for: A one-step multiplex qPCR assay for simultaneous identification and quantification of Leishmania martiniquensis and Leishmania orientalis/Leishmania chancei and detection and quantification of trypanosomatids in clinical samples
Source: Parasite. 2025 Jun 24;32:37. doi: 10.1051/parasite/2025030 (PMC12187069; doi:10.1051/parasite/2025030)
Supplement: Supplementary file 2 — Table S2. Clinical diagnosis in the 69 residual DNA samples: 44 positive (42 for L. martiniquensis and 2 for L. orientalis) samples extracted from clinical specimens of leishmaniasis patients and 25 negative samples using the multiplex qPCR assay. [file parasite-32-37-s2.pdf]

Table S2. Clinical diagnosis in the 69 residual DNA samples: 44 positive (42 for *L. martiniquensis* and 2 for *L. orientalis*) samples extracted from clinical specimens of leishmaniasis patients and 25 negative samples using the multiplex qPCR assay.

Sample	Quantification cycle (Cq)				Result (species or negative)	Parasite burden (parasite equivalent/reaction)
	ITS1-L. mar-HEX	ITS1-L. ori/cha-FAM	ITS1-Tryps- Texas Red	RP-human-Cy5		
LM1	28.26	ND[a]	28.65	26.68	*L. martiniquensis*	0.21
LM2	14.27	ND	13.30	24.63	*L. martiniquensis*	1822.01
LM3	27.42	ND	28.92	24.08	*L. martiniquensis*	0.36
LM4	27.46	ND	28.38	21.56	*L. martiniquensis*	0.35
LM5	27.69	ND	28.51	22.24	*L. martiniquensis*	0.30
LM6	26.75	ND	27.13	24.87	*L. martiniquensis*	0.56
LM7	28.13	ND	27.20	28.44	*L. martiniquensis*	0.23
LM8	28.36	ND	28.71	26.29	*L. martiniquensis*	0.20
LM9	27.85	ND	27.16	22.23	*L. martiniquensis*	0.27
LM10	22.17	ND	21.35	21.29	*L. martiniquensis*	10.88
LM11	27.94	ND	28.35	23.50	*L. martiniquensis*	0.26
LM12	27.52	ND	28.69	25.01	*L. martiniquensis*	0.34
LM13	27.88	ND	27.00	22.91	*L. martiniquensis*	0.27
LM14	27.82	ND	28.71	23.85	*L. martiniquensis*	0.28
LM15	28.29	ND	29.02	26.18	*L. martiniquensis*	0.21
LM16	27.70	ND	28.18	24.29	*L. martiniquensis*	0.30
LM17	27.52	ND	28.16	28.05	*L. martiniquensis*	0.34
LM18	26.80	ND	27.92	23.74	*L. martiniquensis*	0.54
LM19	26.41	ND	27.14	23.44	*L. martiniquensis*	0.70
LM20	26.66	ND	27.94	28.48	*L. martiniquensis*	0.59
LM21	22.11	ND	21.36	21.98	*L. martiniquensis*	11.31
LM22	20.26	ND	19.44	28.25	*L. martiniquensis*	37.51
LM23	28.05	ND	28.21	28.36	*L. martiniquensis*	0.24
LM24	27.32	ND	28.00	26.25	*L. martiniquensis*	0.39
LM25	27.34	ND	28.93	25.48	*L. martiniquensis*	0.38
LM26	28.24	ND	29.03	27.79	*L. martiniquensis*	0.21
LM27	27.86	ND	29.11	28.04	*L. martiniquensis*	0.27
LM28	27.85	ND	28.15	28.40	*L. martiniquensis*	0.27
LM29	28.30	ND	28.32	27.24	*L. martiniquensis*	0.20
LM30	27.61	ND	26.16	24.79	*L. martiniquensis*	0.32
LM31	28.02	ND	28.84	22.49	*L. martiniquensis*	0.24
LM32	27.63	ND	29.72	23.43	*L. martiniquensis*	0.32
LM33	28.30	ND	27.71	21.99	*L. martiniquensis*	0.20

LM34	27.45	ND	27.62	22.75	*L. martiniquensis*	0.35
LM35	28.28	ND	29.08	25.60	*L. martiniquensis*	0.21
LM36	28.12	ND	29.40	27.48	*L. martiniquensis*	0.23
LM37	15.88	ND	15.13	19.52	*L. martiniquensis*	641.63
LM38	27.95	ND	29.16	23.62	*L. martiniquensis*	0.26
LM39	26.73	ND	29.27	21.81	*L. martiniquensis*	0.57
LM40	26.81	ND	29.31	25.62	*L. martiniquensis*	0.54
LM41	27.64	ND	28.39	23.97	*L. martiniquensis*	0.31
LM42	27.45	ND	29.78	24.08	*L. martiniquensis*	0.36
LO1	ND	23.31	24.38	25.40	*L. orientalis*	23.33
LO2	ND	22.13	23.04	25.19	*L. orientalis*	50.84
N1	ND	ND	ND	25.07	Negative	ND
N2	ND	ND	ND	26.70	Negative	ND
N3	ND	ND	ND	25.59	Negative	ND
N4	ND	ND	ND	24.33	Negative	ND
N5	ND	ND	ND	29.44	Negative	ND
N6	ND	ND	ND	26.82	Negative	ND
N7	ND	ND	ND	24.79	Negative	ND
N8	ND	ND	ND	28.18	Negative	ND
N9	ND	ND	ND	24.61	Negative	ND
N10	ND	ND	ND	24.33	Negative	ND
N11	ND	ND	ND	21.74	Negative	ND
N12	ND	ND	ND	26.57	Negative	ND
N13	ND	ND	ND	23.87	Negative	ND
N14	ND	ND	ND	23.64	Negative	ND
N15	ND	ND	ND	23.61	Negative	ND
N16	ND	ND	ND	23.98	Negative	ND
N17	ND	ND	ND	23.69	Negative	ND
N18	ND	ND	ND	23.71	Negative	ND
N19	ND	ND	ND	23.87	Negative	ND
N20	ND	ND	ND	23.74	Negative	ND
N21	ND	ND	ND	23.95	Negative	ND
N22	ND	ND	ND	23.73	Negative	ND
N23	ND	ND	ND	23.81	Negative	ND
N24	ND	ND	ND	23.67	Negative	ND
N25	ND	ND	ND	23.80	Negative	ND

[a]ND = Not detect

Calculation of parasite burden

Parasite burden (parasite equivalent/reaction) = [(DNA quantity (fg/μL) × parasite concentration (parasites))/10^7 fg/μL] × 2

Where:

- DNA quantity (fg/μL) is calculated from the standard curve based on the Cq value

- Parasite concentration = number of parasites in the reference DNA (e.g., 1.5×10^5 parasites in 10^7 fg/μL for *L. martiniquensis*)

- The multiplier ×2 accounts for the 2 μL of DNA template used per qPCR reaction
